# Brain‐Derived Neurotrophic Factor (BDNF) and Sex Differences in Metabolic Regulation

**DOI:** 10.1111/jnc.70245

**Published:** 2025-10-06

**Authors:** Ariane M. Zanesco, Licio A. Velloso

**Affiliations:** ^1^ Laboratory of Cell Signaling‐Obesity and Comorbidities Research Center University of Campinas Campinas Brazil; ^2^ National Institute of Science and Technology on Neuroimmunomodulation Rio de Janeiro Brazil

**Keywords:** BDNF, diabetes, leptin, obesity, reproduction

## Abstract

For decades, most experimental studies were conducted using male rodents as models, and the results obtained in several distinct fields of medical and biological research were regarded as valid for both males and females. However, as evidence progressively challenged this concept by unveiling phenotypes that are regulated according to a pattern of sexual dimorphism, many studies were undertaken to identify the mechanisms driving sex‐specific characteristics. In this context, hypothalamic brain‐derived neurotrophic factor emerged as an important player regulating metabolism according to a sexual dimorphic pattern. Here, we performed a narrative review that puts together the main pieces of evidence showing how brain‐derived neurotrophic factor is involved in metabolic sexual dimorphism. The accumulated data in this field has uncovered important aspects of the physiological and pathological control of metabolic sex‐specific functions and has placed hypothalamic brain‐derived neurotrophic factor as a potential target for interventions aimed at mitigating metabolic abnormalities that affect differently females and males.

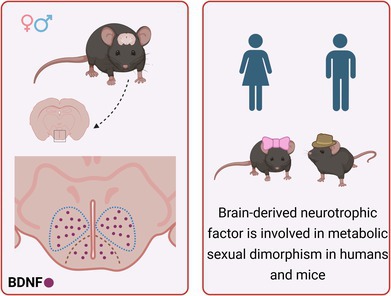

## Graphical abstract

Brain‐derived neurotrophic factor (BDNF) is expressed in the hypothalamus of both female and male mice (left‐hand side of the panel); however, it exerts distinct functions in either sex, explaining some sexually dimorphic phenotypes in mice and humans (right‐hand side of the panel).

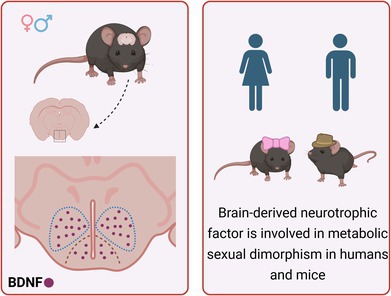

**Abbreviations**
3V: third ventricle
AgRP: agouti‐related protein
ARC: arcuate nucleus of the hypothalamus
BDNF: brain‐derived neurotrophic factor
BW: body weight
CAMK: Ca2+/calmodulin‐dependent protein kinase
cJUN: Jun proto‐oncogene
db/db: diabetic mice model
DMH: dorsomedial nucleus of the hypothalamus
EE: energy expenditure
ERK: extracellular signal‐regulated kinase
FezF1: FEZ Family Zinc Finger 1
FI: food intake
FOSH: female obesity‐related secondary hypogonadism
GDF15: growth differentiation factor 15
GFRAL: GDNF‐family receptor α‐like
GLP‐1: glucagon‐like peptide‐1
GLP1R: glucagon‐like peptide‐1 receptor
GnRH: gonadotrophin‐releasing hormone
Kiss1: kisspeptin 1
LA: locomotor activity
LH: luteinizing hormone
MBH: mediobasal hypothalamus
MC4R: melanocortin 4 receptor
MOSH: Male obesity‐related secondary hypogonadism
mRNA: messenger RNA
mTOR: mammalian target of rapamycin
NF‐kB: nuclear factor kappa‐B
Nkx2.1: NK2 Homeobox 1
p75^NTR^
: p75 neurotrophin receptor
PCOS: polycystic ovary syndrome
PKC: protein kinase C
POMC: pro‐opiomelanocortin
PVH: paraventricular nucleus of the hypothalamus
SF1: Steroidogenic factor 1
TrkB: tyrosine receptor kinase B
VMH: ventromedial nucleus of the hypothalamus

## 1 Introduction

The accelerated increase in obesity rates over the last 50 years has provided the pathophysiological ground for the subsequent increase in the rates of several other metabolic and cardiovascular conditions that threaten longevity and life quality on a worldwide scale (Salama et al. 2023) (Piché et al. 2020) (Ferreira et al. 2024). The increased consumption of highly palatable and energy‐dense foods, associated with a sedentary lifestyle, is the main reason behind the obesity pandemic (Shim 2025) (Hopkins and Blundell 2016). Considering that both factors could be modified by the will of the affected subjects, it is intuitive to believe that behavioral approaches, aimed at reducing caloric intake and stimulating the regular practice of exercise, should be successful in controlling obesity. Nevertheless, as shown by studies that evaluated the long‐term outcomes of behavioral approaches to treat obesity, most patients reduce body mass during the first 6 months of intervention, only to regain body mass thereafter, frequently to a level greater than the one they started the intervention (LeBlanc et al. 2018). Long‐term follow‐up of patients submitted to bariatric surgery also shows some regain of body mass (Carlsson et al. 2025), and this is also the case for patients undergoing body mass reduction during the treatment with the agonists of the glucagon‐like peptide‐1 receptor (GLP1R) (Wilding et al. 2022). Thus, therapeutic refractoriness and high rates of recurrence are important problems faced in the management of obesity, and in order to obtain consistent advancement in this field, it is important to define the mechanisms behind these outcomes. As the response to therapy is variable among patients, it has been proposed that defining the phenotypes that are associated with a better outcome for each of the therapeutic interventions available could improve the overall results achieved (Acosta et al. 2021). However, the capacity to precisely define the patients' phenotypes has proven awkward, which explains criticism of this approach (Hocking and Sumithran 2023) (Kachmar et al. 2025).

Although defining eating patterns and behavioral phenotypes associated with distinct responses to obesity therapeutic interventions may be a difficult task, there are other phenotypes that could be more easily determined, being sex one of the most straightforward. Even though obesity‐associated metabolic and cardiovascular diseases represent serious health problems for both women and men, there are important sex differences regarding the prevalence and the clinical aspects of these diseases that could be explored in the context of defining optimized sex‐specific preventive and therapeutic strategies. In most parts of the world, obesity is more prevalent in women than in men (Cooper et al. 2021); however, men respond better to behavioral interventions aimed at reducing food intake and sedentariness, whereas women respond better to pharmacological treatment (Susanto et al. 2022) (Verma et al. 2024). Conversely, diabetes is more prevalent in men with obesity, whereas women frequently have more cardiovascular and renal complications attributed to the poor control of diabetes (Kautzky‐Willer et al. 2023). As a rule, most studies exploring the mechanisms behind sexual dimorphism in obesity‐associated metabolic and cardiovascular diseases are focused on the roles played by sex hormones.

In obesity, there is a bidirectional association of increased adiposity and sex hormones; thus, the development of obesity can affect the levels and function of sex hormones, whereas primary changes in sex hormones can predispose one to obesity (Ylli et al. 2000). In men, obesity is frequently associated with low levels of testosterone; in a meta‐analysis that included almost 20 thousand patients with obesity, prevalence of hypogonadism was up to 51% depending on whether low total testosterone or low free testosterone was used to define hypogonadism (van Hulsteijn et al. 2020). This condition is known as male obesity‐related secondary hypogonadism (MOSH) (Eng et al. 2024), and a recent meta‐analysis revealed the benefits of testosterone replacement, leading to improvements in erectile dysfunction and life quality (Hudson et al. 2023). In addition, in males that are primarily hypogonadal, obesity develops in up to 50%, which is a much greater frequency than in non‐hypogonadal men (Camacho et al. 2013).

In women, the association of hypogonadism with obesity is not as explored as in men. In fact, only recently it was proposed the existence of a female obesity‐related secondary hypogonadism (FOSH) (Eng et al. 2024). For decades, the main phenotypic feature studied in women with obesity was polycystic ovary syndrome (PCOS) (Pagán et al. 2006). However, this condition cannot be taken as the female counterpart of MOSH, as the levels of gonadotrophins are increased as opposed to the reduced levels found in men (Eng et al. 2024). In many women with obesity without PCOS, there is a negative impact on LH pulse amplitude rather than pulse frequency (Al‐Safi et al. 2015). In addition, there is greater estradiol‐induced negative feedback on the hypothalamic–pituitary‐gonadal axis, resulting in reduced ovarian stimulation and lower luteal progesterone levels with increased estradiol‐induced negative feedback.

Despite the consistent advance obtained in this field, it is currently unknown what the main determinants of MOSH and FOSH are. As the brain plays a central role in the regulation of food intake, energy expenditure, and reproduction, it is possible that brain factors play important roles in this scenario. During the last 10 years, studies have provided evidence suggesting that brain‐derived neurotrophic factor (BDNF) could be involved or associated with sexual dimorphism in conditions such as cognition, response to stress, response to pain, and metabolism (Lionetti and Recchia 2024) (Melgar‐Locatelli et al. 2024) (Dedek et al. 2022). Here, we performed a narrative review that explores the association of BDNF on metabolic sexual dimorphism.

## 2 A General View of BDNF

**FIGURE 1:**
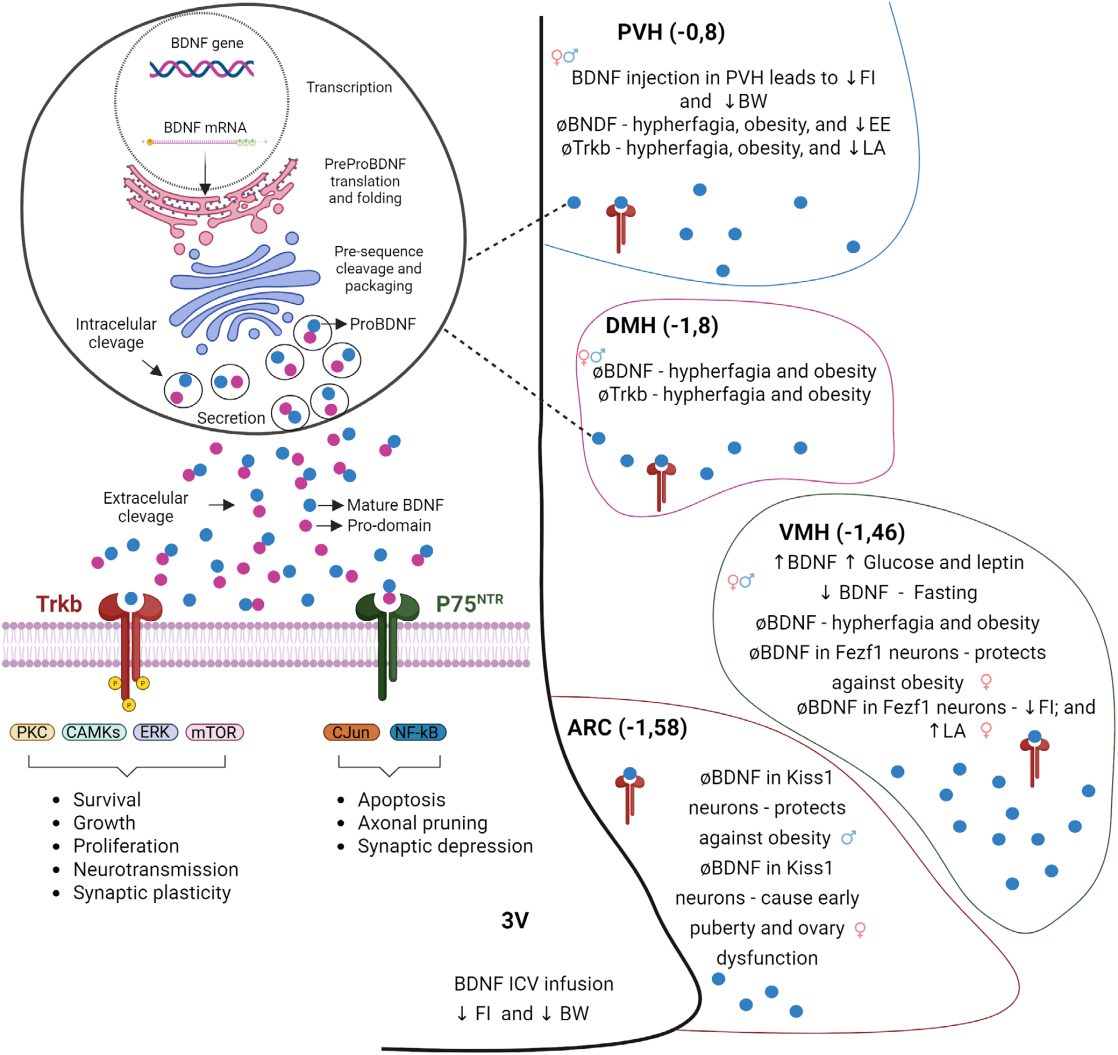
*Brain‐*derived neurotrophic factor. The general aspects of synthesis and actions of brain‐derived neurotrophic factor are presented on the left‐hand side of the panel. The outcomes of increasing or decreasing the actions of brain‐derived neurotrophic factor in the main regions of the hypothalamus are presented on the right‐hand side of the panel. BDNF, brain‐derived neurotrophic factor; TrkB, tyrosine receptor kinase B; p75^NTR^, p75 neurotrophin receptor; PKC, protein kinase C; CAMK, Ca^2+^/calmodulin‐dependent protein kinase; ERK, extracellular signal‐regulated kinase; mTOR, mammalian target of rapamycin; cJUN, jun proto‐oncogene; NF‐kB, nuclear factor kappa‐B; PVH, paraventricular nucleus of the hypothalamus; DMH, dorsomedial nucleus of the hypothalamus; VMH, ventromedial nucleus of the hypothalamus; ARC, arcuate nucleus of the hypothalamus; BW, body weight; FI, food intake; EE, energy expenditure; LA, Locomotor activity; FezF1, FEZ Family Zinc Finger 1; 3V, third ventricle.

BDNF is a peptide coded by a cognate gene located in the short arm of human chromosome 11 (chromosome 2 in mice) (Jones and Reichardt 1990). The nascent 247‐amino acid protein is enzymatically processed to produce the mature and biologically active 138‐amino acid peptide that acts through two distinct membrane receptors, TrkB and p75^NTR^ (Fu et al. 2014). TrkB signals through PKC, CAMKs, ERK, and mTOR to control growth, survival, proliferation, neurotransmission, and synaptic plasticity; p75^NTR^ acts through cJUN and NF‐kB to regulate apoptosis, axonal pruning, and synapsis depression (Figure 1, left‐hand side of the panel). BDNF belongs to the neurotrophin family of growth factors and is related to the canonical neurotrophin, nerve growth factor (McDonald and Hendrickson 1993). Most studies in this field have explored the actions of BDNF in the brain; however, it is also present in peripheral nerves and non‐neural tissues, such as the kidney, ovaries, skeletal muscle, and prostate (Raznahan et al. 2009) (Lommatzsch et al. 2005). In the brain, BDNF is involved in the growth, development, differentiation, and maturation of neurons, as well as in synapsis formation; the proper function of BDNF is implicated in the optimal development and maintenance of learning, memory, and cognition (Hofer and Barde 1988) (Klöcker et al. 2000) (Xu et al. 2000) (Phillips et al. 1991). There is both experimental and clinical evidence demonstrating that the abnormal function of BDNF is associated with neurological conditions such as Alzheimer's Disease, Parkinson's Disease, and Huntington's Disease (Phillips et al. 1991) (Zuccato et al. 2001) (Howells et al. 2000). Nevertheless, over the last 30 years, studies have provided consistent evidence linking BDNF with metabolic conditions such as diabetes, obesity, atherosclerosis, and sarcopenia (Ono et al. 1997) (Wiegand et al. 2004) (Friedel et al. 2005) (Yeo et al. 2004) (Bi et al. 2020) (Pratt et al. 2025).

## 3 BDNF And the Control of Metabolism

On early studies related to the potential involvement of BDNF in metabolic conditions, the focus was on diabetes‐associated neuropathy (Apfel 1999). It was shown that the tissue levels of neurotrophins, including BDNF, were reduced in this condition, and based on that, it was proposed that using exogenous neurotrophins could be a promising strategy to treat or mitigate diabetes‐associated neuropathy and other types of neuropathies (Apfel 1999) (Norata et al. 2025). However, despite encouraging results in pre‐clinical trials, there are no substances of this class currently approved for medical use in any type of neuropathy (Norata et al. 2025).

The first experimental evidence that BDNF could provide metabolic control in diabetes was published in 1997 (Ono et al. 1997). In that study, the authors employed db/db mice to test the hypothesis that BDNF could mitigate hyperglycemia. The db/db mice are natural mutants with a loss of function for the leptin receptor gene. They spontaneously develop obesity and diabetes and are frequently employed in experimental studies in this field (Tartaglia et al. 1995). BDNF was injected subcutaneously, 5 days per week at the dose of 20 mg/kg. At baseline, mice presented blood glucose levels superior to 500 mg/dL. As early as 5 days after the beginning of the intervention, blood glucose levels were significantly reduced. Moreover, insulin levels reduced, and glucose tolerance improved, suggesting that the action of BDNF was mostly related to an improvement in insulin sensitivity (Ono et al. 1997).

Another important advance in the field was obtained by the demonstration that a genetic disruption of a regulatory region of the Bdnf gene that reduced BDNF expression in mice resulted in obesity and diabetes (Sha et al. 2007). This was rapidly followed by the identification of human variants of the BDNF gene that were associated with diabetes and obesity (Ng et al. 2010) (Han et al. 2013). However, the greatest evidence for the role of BDNF in the regulation of metabolism came from the studies with genetic ablation of the Bdnf gene in mice. In an early study that produced mice with a homozygous deletion of the Bdnf gene, it was reported that mice were unviable, dying a few days after birth (Patterson et al. 1996). Nevertheless, when heterozygous Bdnf knockout mice were studied, important metabolic phenotypes were identified (Kernie et al. 2000). First, it was shown that mutant mice were hyperphagic and developed obesity at the age of 14 weeks. Interestingly, despite the development of obesity, mice were hyperactive; however, when locomotor activity was analyzed against body mass, it resulted in a significantly inverse correlation, suggesting that locomotor activity could be related to the search for food, and once obesity developed, activity reduced (Kernie et al. 2000). Mutant mice were also hyperleptinemic and hyperinsulinemic, despite normal baseline levels of glucose, suggesting that the pancreatic beta‐cells were still capable of responding properly to the systemic demand generated by insulin resistance (Kernie et al. 2000). Finally, as the functions most affected by the heterozygous disruption of the Bdnf gene were related to actions exerted by hypothalamic neurons, authors evaluated the hypothalamic expression of Bdnf and its receptor TrkB. It was shown that Bdnf was mostly expressed in the ventromedial and dorsomedial hypothalamus (VMH and DMH, respectively), whereas TrkB was widely expressed throughout the hypothalamus (Kernie et al. 2000). These results fostered interest in understanding the roles of BDNF in the hypothalamus and all its potential implications on the central regulation of metabolism and other systemic functions exerted by hypothalamic neurons.

Following the identification of leptin in the mid 1990s (Zhang et al. 1994), rapid progress was achieved in the characterization of hypothalamic neurocircuits that regulate food intake, energy expenditure, and several other functions exerted by the hypothalamic neurons. Currently, it is known that AgRP and POMC neurons present in the arcuate nucleus act as first‐order neurons sensing systemic signals that indicate the energy stored in the body (Cavadas et al. 2016). These signals are constituted by hormones, such as leptin, insulin, GLP1, and ghrelin, and nutrients that are present in the bloodstream and cross the permissive blood–brain barrier present in the median eminence–mediobasal hypothalamus (MBH) interface (Haddad‐Tóvolli et al. 2017) to act on AgRP and POMC neurons. In response to these signals, MBH neurons connect to a complex hypothalamic and extrahypothalamic network to regulate systemic functions.

An article published in 2003 by Louis Reichardt and coworkers provided the first detailed evaluation of hypothalamic BDNF (Xu et al. 2003). First, they confirmed that the VMH was the hypothalamic region expressing the greatest amount of BDNF, and that caloric deprivation promoted a reduction of Bdnf expression. In addition, it was shown that a genetic modification of the Trkb gene that resulted in reduced tyrosine kinase activity of the receptor resulted in obesity due to increased caloric intake. Using an agouti mutant, it was shown that the inhibition of signal transduction through MC4R promoted a reduction of VMH BDNF, whereas the intracerebroventricular injection of an agonist of MC4R increased BDNF. Other genetic strategies were used to further explore the roles of BDNF in the hypothalamus; thus, the knockdown of Bdnf in SF1 neurons promoted hyperphagia and obesity (Yang et al. 2016); the knockout of Bdnf in Nkx2.1 neurons resulted in mild obesity (Yang et al. 2016); and in a study using the Cre‐Lox system to further explore the roles of BDNF in the VMH, it was identified an ARC‐VMH‐perimesencephalic trigeminal area circuit that controls caloric intake by gating motor sequences of feeding (Kosse et al. 2024), and a leptin‐dependent neurocircuit that provides sympathetic innervation to the adipose tissue.

Inhibiting BDNF in specific regions of the hypothalamus was yet another approach used to explore its roles in the hypothalamus. This was performed using site‐specific injections with adeno‐associated virus constructed with an inhibitory sequence of the Bdnf gene (Unger et al. 2007). The specific deletion of Bdnf in the VMH and DMH of adult male mice resulted in hyperphagic behavior and obesity without affecting energy expenditure (Unger et al. 2007). The deletion of the Bdnf gene specifically in the PVH led to marked hyperphagia and severe obesity (An et al. 2015). In addition, a population of BDNF‐positive cells located in the medial nucleus of the tractus solitarius was identified as downstream of GLP1R and GFRAL (GDNF‐family receptor α‐like) neurons. Using transgenic mice, they confirmed that these BDNF‐positive neurons are required for the weight‐reducing actions of GDF15 (growth differentiation factor 15) and GLP‐1 (glucagon‐like peptide‐1) (Feetham et al. 2024). Thus, taking together the results of all these studies, there is consistent experimental evidence indicating that hypothalamic BDNF plays an important role in the regulation of whole‐body metabolism (Figure 1, right‐hand side of the panel).

## 4 Evidence for the Actions of BDNF on Metabolic Sexual Dimorphism

The first evidence suggesting that BDNF could be involved in sexual dimorphism came from studies on the characterization of the response to stress (Zuena et al. 2008) (Lin et al. 2009). In male rats that were born from mothers submitted to gestational restrain stress, the levels of hippocampal BDNF were increased, and this was accompanied by the development of an anxiety‐like behavior. Conversely, in females raised under the same conditions, there was a phenotype of improved learning and no changes in hippocampal BDNF (Zuena et al. 2008). In a study that explored the impact of acute and chronic stress, it was shown that there was a reduction and an increase in brain BDNF in females exposed to acute and chronic stress, respectively. In males, BDNF changes were detected neither in acute nor in chronic stress (Lin et al. 2009).

An important advance in the field was the demonstration that Bdnf is co‐localized with the transcripts for estrogen receptor‐alpha in the VMH (Blurton‐Jones et al. 2004). Currently, it is known that certain hypothalamic regions are sexually dimorphic, a phenomenon mostly attributed to the differential expression of sex steroid receptors, which exert distinct physiological effects in males and females (Donato et al. 2010). From an evolutionary perspective, sex‐related differences in energy regulation and metabolism may have emerged from the distinct biological roles of the sexes, as males traditionally are involved in hunting and foraging, whereas females are primarily responsible for gestation, lactation, and caregiving. Both roles require finely tuned mechanisms for maintaining energy balance to support individual survival and the successful development of the offspring (Liu et al. 2014).

The potential implication of hypothalamic BDNF on the regulation of metabolic sexual dimorphic phenotypes was further supported by a study that used a chemogenetic approach to inhibit Trkb during different periods of embryo development and during the post‐natal period. The study revealed a sexually dimorphic effect of Trkb inhibition on both body weight and hypothalamic expression of genes known to regulate food intake and body mass (Byerly et al. 2013). Another study investigated how peripheral signals and central Bdnf expression were regulated in response to dietary interventions. Several differences were identified between female and male VMH BDNF expression; VMH Bdnf expression was down‐regulated by dietary intervention in male rats, whereas in female rats under equal dietary disruption, VMH Bdnf expression was stable (Liu et al. 2014).

Furthermore, another piece of evidence connecting hypothalamic BDNF with sexual dimorphism was provided in a study that evaluated Bdnf expression during the ovarian cycle. The study showed that VMH BDNF mRNA is dynamically regulated across the ovarian cycle with an increase at the estrous phase following the estradiol peak, and this coincided with the decline in feeding at this phase of the ovarian cycle in intact female rats (Zhu et al. 2013). This mechanism is very similar to what happens with fertile women; in the luteal phase, the levels of plasma BDNF are significantly greater than at the follicular phase. BDNF increased from the early follicular phase up to Day 14 of the cycle, reaching a pre‐ovulatory peak, similar to estrogen levels, but the greatest peak occurs at the luteal phase. Conversely, menopausal women show a significant decrease in the plasmatic levels of BDNF compared with fertile women (Begliuomini et al. 2007). Thus, age is a crucial factor to consider when comparing the levels of BDNF in women and men. A study investigating the relationship between BDNF levels and factors such as age, gender, body mass index, and body weight concluded that females exhibited lower levels of blood BDNF compared to males, without considering age as a covariant (Pillai et al. 2012). However, when the levels of BDNF are analyzed in younger volunteers, women presented higher levels of BDNF in serum compared to men (Glud et al. 2019). These findings reinforce the influence of sex hormone levels on BDNF circulation.

Based on the findings that BDNF and Trkb act in a dimorphic way in the control of metabolism, a recent study selectively deleted Trkb in the PVH of female and male mice and showed opposed results regarding the control of food intake: suppression in males and increase in females (An et al. 2015). This phenotype was reproduced using a different approach with chemogenetic modulation of TrkB activity. Additionally, the specific activation of BDNF‐expressing PVH neurons using chemogenetic approaches rapidly decreased normal nocturnal feeding and fasting‐induced feeding in male and female mice. At thermoneutral temperatures, acute activation also rapidly increased adaptive thermogenesis, increased core body temperature, increased locomotion, increased energy expenditure, and decreased respiratory exchange ratio in male and female mice. Taken together, these results provide strong experimental evidence supporting the role of hypothalamic BDNF in the sex‐specific aspects of metabolic regulation (Wu and Xu 2022).

## 5 Hypothalamic BDNF and the Control of Reproduction

The function of hypothalamic BDNF on sexual dimorphism is not restricted to metabolism but also to the control of reproduction. TrkB is present in key neurons that express gonadotrophin‐releasing hormone (GnRH), indicating that BDNF is essential for both the development and function of the GnRH secretory system during the mice embryonic period (An et al. 2015). In addition, we have recently shown that Bdnf is expressed in the arcuate nucleus Kiss1 neurons in a sex and temporal‐dependent way, which may impact differently the development of hypothalamic neurocircuits that regulate reproduction in females and males (Zanesco et al. 2024).

The actions of BDNF in reproduction extend to the regulation of oogenesis, follicle recruitment, germ cell survival, and nuclear and cytoplasmic maturation, suggesting that BDNF–TrkB signaling is important in embryo development, implantation survival, and placental function (Chow et al. 2020). Moreover, in women, differences in blood levels of BDNF are related to reproductive disorders, such as endometriosis, PCOS, functional hypothalamic amenorrhea, and early ovarian syndrome (Russo et al. 2012). As previously mentioned, the blood levels of BDNF are influenced by estradiol fluctuation during the menstrual cycle; in addition, BDNF levels also follow a circadian rhythm declining over the course of the day and inversely correlate with plasma cortisol levels (Pluchino et al. 2009).

There is greater evidence of BDNF–TrkB functions in the female reproductive tract as compared to the male. In the ovary, BDNF and TrkB are expressed in mesenchymal cells before follicle assembly as well as in granulosa cells, thecal cells, and oocytes. In the ovarian follicles, BDNF, acting through TrkB, supports early follicle development. In addition, BDNF contributes to the cytoplasmic and nuclear maturation of the oocyte. We have recently shown that the depletion of BDNF in Kiss1 neurons is responsible for promoting follicle apoptosis, increasing the number of atretic follicles in the ovary of fertile females, indicating the importance of BDNF produced by Kiss1 neurons in the survival and maintenance of the follicles (Zanesco et al. 2024).

Both BDNF and TrkB are present in human and rodent testis; however, little is known about their functions in the male reproductive system. Interestingly, BDNF is expressed during the embryonic period and the beginning of life in rat Sertoli cells, which indicates that BDNF may induce the progression of testicular development. In adult human testis and adult mice testis, BDNF mRNA is expressed in Sertoli and Leydig cells. In concert, BDNF mRNA and protein are present in mature ejaculated human spermatozoa from fertile and infertile men (Zheng et al. 2011). Thus, the characterization of BDNF expression and function in the reproductive tract is still an incipient field of research, and further studies are needed.

## 6 Limitations of the Studies in This Field

We have identified some limitations in the original studies published in this field, and by listing them, we hope to provide guidance for studies performed in the future:

- Most studies evaluated males, only;
- Most studies evaluated cell‐nonspecific actions of BDNF and TrkB in the hypothalamus;
- Very little has been performed regarding the actions of hypothalamic BDNF in the control of reproduction.

## 7 Conclusions

BDNF has emerged as an important player in the regulation of sexual dimorphic functions related to metabolism and reproduction. The fine characterization of its roles in these functions could expand the understanding of how metabolism and reproduction are integrated; how genetic factors could affect the pathophysiology of metabolic conditions that have different presentations in females and males; and how we could advance in the development of therapeutic interventions for metabolic diseases that are tailored taking into account sexual differences.

## Author Contributions

**Ariane M. Zanesco:** conceptualization, writing – original draft, writing – review and editing. **Licio A. Velloso:** conceptualization, funding acquisition, writing – original draft, writing – review and editing, validation, visualization, supervision.

## Consent

Informed consent was achieved for all subjects, and the experiments were approved by the local ethics committee.

## Conflicts of Interest

The authors declare no conflicts of interest.

## Peer Review

The peer review history for this article is available at https://www.webofscience.com/api/gateway/wos/peer‐review/10.1111/jnc.70245.

## Data Availability

The authors have nothing to report.
